# Preventing mucus plugs in COPD: a potential role for erdosteine

**DOI:** 10.1183/13993003.01274-2026

**Published:** 2026-08-27

**Authors:** Peter M.A. Calverley

**Affiliations:** Institute of Life Course and Medical Sciences, University of Liverpool, Liverpool, UK

## Abstract

*To the Editor*:

## Abstract 2

**Mucus plugging is an important component of clinical deterioration in COPD. Erdosteine, a muco-active agent used early in the evolution of COPD, reduces exacerbations and may slow plug development.** https://bit.ly/4ePJlf9

*To the Editor*:

I read with great interest the excellent review of the role of mucus plugging in obstructive lung disease by Fahy [1]. He has provided us with a comprehensive and thoughtful overview of the importance of mucus plugging in asthma and COPD, and has highlighted the rapid progress being made in our knowledge about the nature and distribution of mucus plugs in the airways. Understanding the processes involved in plug evolution and resolution is going to be crucial for rational therapy, as outlined in the review. Muco-active agents such as *N*-acetylcysteine have proven to be disappointing for treating established plugs, but as is noted, they may have some effect on exacerbations of disease.

One agent not discussed in the review is erdosteine, a thiol-containing pro-drug currently licensed for the prevention of COPD exacerbations in Europe and around the world, but not yet in the USA. This agent, which has muco-active, antioxidant and antibiotic-enhancing properties, appears to be more effective than other muco-active drugs [2] and has been predominantly studied in COPD rather than asthma patients. I was involved in the steering committee of the RESTORE study, a 1-year trial of twice daily erdosteine in moderate/severe COPD patients, the results of which were published in the *European Respiratory Journal* [3]. Erdosteine treatment significantly decreased the number and duration of exacerbations, despite 73% of participants taking inhaled corticosteroids. The treatment effect was most marked in patients with less severe airflow obstruction [4]. These findings are in keeping with the data summarised by Fahy [1], where muco-active drugs did not clear established plugs in more severe COPD, but our results suggest that modifying the properties of mucus in less severe disease can lead to clinical benefit. More recent “real world” data supports a sustained effect of erdosteine in COPD exacerbation prevention [5].

Exacerbations of COPD are associated with increases in MUC5AC in experimental and naturally occurring viral infections [6], making changes in the physical properties of the mucus an important therapeutic goal. COPD exacerbations contribute to the accelerated lung function decline that is characteristic of COPD [7], a process accompanied by changes in both the biophysical properties of mucus and the airway microbiome [8]. As outlined in the conclusions of the review, multiple approaches will likely be needed to prevent the formation and aid the resolution of mucus plugging. In this regard, drugs such as erdosteine can play a role and merit further investigation using the exciting tools now being developed to address this important problem.
